# Poorer Cognitive Performance in Patients with Essential Tremor-Parkinson’s Disease vs. Patients with Parkinson’s Disease

**DOI:** 10.3389/fneur.2015.00106

**Published:** 2015-05-18

**Authors:** Elan D. Louis, Brittany Rohl, Kathleen Collins, Stephane Cosentino

**Affiliations:** ^1^Division of Movement Disorders, Department of Neurology, Yale School of Medicine, Yale University, New Haven, CT, USA; ^2^Department of Chronic Disease Epidemiology, Yale School of Public Health, Yale University, New Haven, CT, USA; ^3^Department of Neurology, College of Physicians and Surgeons, Columbia University, New York, NY, USA; ^4^Taub Institute for Research on Alzheimer’s Disease and the Aging Brain, College of Physicians and Surgeons, Columbia University, New York, NY, USA

**Keywords:** essential tremor, Parkinson’s disease, cognition, clinical, dementia

## Abstract

**Background:**

Patients with essential tremor (ET) seem to be at increased risk of developing Parkinson’s disease (PD). Surprisingly, little has been written about this clinical entity, ET-PD. Cognitive dysfunction is a well-known feature of PD, and can also be an issue in patients with ET. Whether the presence of the combined diagnosis, ET-PD, is associated with additive cognitive effects as compared with PD has not been studied.

**Methods:**

Thirty ET-PD patients and 53 age-matched PD patients were enrolled in a clinical-epidemiological study. Two cognitive screens, the Telephone Interview for Cognitive Status (TICS, score = 0–41) and Folstein Mini-Mental State Examination (MMSE; range 0–30), were administered.

**Results:**

The MMSE score was lower in ET-PD than PD [26.5 ± 3.1 (median 28.0) vs. 28.4 ± 2.2 (median 29.0), *p* = 0.001]. The TICS score was lower in ET-PD than PD [31.7 ± 3.9 (32.0) vs. 35.0 ± 2.0 (35.0), *p* < 0.001]. Subscores of these tests that related to orientation (*p* < 0.001), language (*p* < 0.001), and working memory (*p* = 0.001) were lower in ET-PD than PD, whereas the delayed memory subscore was only marginally lower in ET-PD than PD (*p* = 0.06), and the two groups did not differ with respect to the motor/construction subscore (*p* = 0.22). Both global cognitive scores were inversely correlated with disease duration (for MMSE score, Spearman’s *r* = −0.46, *p* < 0.001; for TICS score, Spearman’s *r* = −0.53, *p* < 0.001).

**Conclusion:**

The combined diagnosis, ET-PD, seemed to be associated with additive cognitive effects as compared with PD alone.

## Introduction

Patients with essential tremor (ET) seem to be at increased risk of developing Parkinson’s disease (PD) (1). Prospective, longitudinal, epidemiological data estimate a four- to five-fold increased risk (1).

Despite this association, surprisingly, little has been written about this clinical entity, “ET-PD,” with only two small case series [*n* = 22 (2), *n* = 53 (3)], both based on retrospective chart review. The cognitive features of this combination disorder have not been studied, as prior reports focused on tremor and other motor features.

Cognitive dysfunction is a well-known feature of PD (4). Recent work indicates that cognitive dysfunction may also be an issue in patients with ET (5), although data are limited. The types of cognitive deficits are not dissimilar in the two disorders, with executive dysfunction and deficits in memory being prominent (5). Whether the presence of the combined diagnosis is associated with additive cognitive effects is not known, but seems plausible.

As part of a prospective, clinical–epidemiological study, we performed a standardized, structured clinical evaluation of 30 ET-PD patients, comparing them to 53 age-matched PD patients. As part of that evaluation, patients underwent several cognitive screens. The goal of these analyses was to compare the cognitive performance in these two patient groups, with the *a priori* hypothesis that ET-PD patients would have poorer performance than their counterparts with PD alone.

## Materials and Methods

### Participants

Participants were enrolled in a clinical–epidemiological study of movement disorders at the Neurological Institute, Columbia University Medical Center (CUMC) (2009–2014) (6). The study assessed the role of environmental toxins in disease etiology; it also evaluated a wide range of clinical features across movement disorders. ET-PD and PD patients seen in the most recent 5 years were identified from a computerized billing database at the Center for Parkinson’s Disease and Other Movement Disorders at the Institute. Each patient had received a diagnosis of PD or ET-PD from their treating neurologist at the Institute. One of the authors (Elan D. Louis) reviewed the office records of identified patients; those with diagnoses of or physical signs consistent with other movement disorders were excluded. During the review, the most recent Hoehn and Yahr score (7), daily dose (milligrams) of levodopa, and other data were recorded.

The CUMC Internal Review Board approved study procedures. Signed informed consent was obtained upon enrollment.

### Study evaluation

During the initial enrollment telephone call, the 10-min Telephone Interview for Cognitive Status [TICS, score = 0–41 (no impairment)] was administered to all participants (8), although no participants were excluded based on their score. An in-person clinical assessment was then performed, during which a trained research assistant administered a series of structured questionnaires, which elicited data on (1) demographic variables, (2) general medical health (Cumulative Illness Rating Scale score (9) [range = 0–42 (maximum)], total number of prescription medications), (3) disease duration (e.g., duration of first motor symptoms in PD and duration of initial action tremor in ET-PD), (4) medication use [taking medication to treat tremor (yes vs. no)], and (5) additional variables of interest (e.g., age of symptom onset). The Center for Epidemiological Studies Depression Scale (CESD-10) was also administered; this is a self-report, 10-item screening questionnaire for depressive symptoms [range = 0–30 (greater depressive symptoms)] (10). To further evaluate cognition, the research assistant administered the Folstein Mini-Mental State Examination [MMSE, range 0–30 (no impairment) (11)].

A videotaped neurological examination was performed on ET-PD patients. This included one test for postural tremor and five for kinetic tremor (e.g., pouring, drinking) performed with each arm (12 tests total). A neurologist specializing in movement disorders (Elan D. Louis) used a reliable and valid clinical rating scale, the Washington Heights-Inwood Genetic Study of ET (WHIGET) tremor rating scale, to rate postural and kinetic tremor during each test: (0–3). These ratings resulted in a total tremor score (range = 0–36).

### Diagnoses

Each patient had received a diagnosis of PD or ET-PD from their treating movement disorders neurologist at the Institute. In addition, based on office record review, the diagnosis of PD was confirmed (Elan D. Louis) prior to enrollment using published diagnostic criteria, which required the presence of at least two cardinal signs (12). The diagnosis of ET-PD was further reviewed prior to enrollment, and required that (1) the ET diagnosis was present for at least 5 years prior to the PD diagnosis, (2) the initial ET was characterized by moderate or greater amplitude kinetic tremor in the absence of any signs of PD (e.g., rest tremor, bradykinesia), and (3) the initial ET diagnosis occurred in absence of red flags for possible emerging PD (isolated postural tremor without kinetic tremor, unilateral kinetic tremor).

### Final subject selection

Data were available on all 30 enrolled ET-PD patients. These were compared to data on 53 PD patients who had been frequency-matched to the ET-PD patients by age.

### Statistical analyses

Data were analyzed in SPSS (Version 21). Demographical and clinical characteristics of ET-PD and PD patients were compared using chi-squared tests, Fisher’s exact tests, Student’s *t*-tests and, when the variable was not normally distributed, Mann–Whitney tests (Table 1). The MMSE score and the TICS score were not normally distributed (respective Kolmogorov–Smirnov *z* = 2.17, *p* < 0.001 and *z* = 1.53, *p* = 0.02). Therefore, correlations between these scores and other variables were assessed with Spearman’s correlation coefficients. The MMSE was not normally distributed, even after log transformation and other forms of transformation (squaring, cubing). By contrast, after transformation as follows: log10(41 − TICS score), the TICS score was normally distributed (Kolmogorov–Smirnov *z* = 1.08, *p* = 0.19). In a linear regression analysis, we assessed the association between disease duration and transformed TICS score (outcome variable), adjusting for age and education. To explore the possibility of confounding by education, in a linear regression model, we assessed the association between diagnosis and transformed TICS score (outcome variable), adjusting for years education.

**Table 1:** **Demographical and clinical characteristics of participants**.

	PD	ET-PD	Significance
*n*	53	30	
Age (years)	76.5 ± 3.4	77.5 ± 7.6	*p* = 0.51^a^
Female gender	24 (45.3)	11 (36.7)	*p* = 0.45^b^
Non-Hispanic white	48 (90.6)	26 (86.7)	*p* = 0.72^c^
Education (years)	16.5 ± 2.7	15.1 ± 4.4	*p* = 0.12^a^
Disease duration (years)	9.9 ± 12.5 (6.0)	32.3 ± 22.1 (28.0)	*p* < 0.001^d^
CIRS score	8.0 ± 3.4	8.4 ± 3.7	*p* = 0.67^a^
CESD-10 score	9.5 ± 5.3	9.1 ± 5.8	*p* = 0.79^a^
Total tremor score	NA	24.0 ± 5.3	NA
Total number of prescription medications	6.5 ± 2.9	6.3 ± 3.4	*p* = 0.77^a^
Takes medication for ET	NA	22 (73.3)	NA
Number of medications for ET	NA	1.0 ±0.8 (0–3)	NA
Takes medication with GABA-ergic properties	2 (3.8)	8 (26.7)	*p* = 0.004^c^
Takes dopamine agonist	9 (17.0)	4 (13.3)	*p* = 0.76^c^
Takes amantadine	3 (5.7)	2 (6.7)	*p* = 1.00^c^
Takes selegiline	13 (24.5)	2 (6.7)	*p* = 0.04^b^
Takes anticholinergic agent	0 (0.0)	0 (0.0)	*p* = 1.00^b^
Takes carbidopa–levodopa	45 (84.9)	15 (50.0)	*p* = 0.001^b^
Carbidopa–levodopa dose (mg)^e^	337.9 ± 342.1 (200)	486.7 ± 274.8 (300)	*p* = 0.02^d^
Years on carbidopa–levodopa^e^	6.1 ± 4.3	5.9 ± 4.3	*p* = 0.88^d^

*Values are mean ± SD (median or range) or number (percentage)*.

*CESD-10, Center for Epidemiological Studies Depression Scale; CIRS, Cumulative Illness Rating Scale; ET, essential tremor; GABA, gamma aminobutyric acid; mg, milligrams; NA, not applicable; PD, Parkinson’s disease*.

*^a^Student’s *t*-test*.

*^b^Chi-squared test*.

*^c^Fisher’s exact test*.

*^d^Mann–Whitney test*.

*^e^Among participants taking carbidopa–levodopa*.

*NA, not applicable*.

Following previously published guidelines (13, 14), the MMSE was divided into subscores, reflecting different cognitive domains. To add to the robustness of our analyses, MMSE subscores were combined with TICS subscores reflecting the same cognitive domains, except for delayed memory, which is available only from the MMSE. The final subscores were orientation (combined), language (combined), working memory (combined), delayed memory (from the MMSE), and motor/construction (from the MMSE).

## Results

Parkinson’s disease and ET-PD patients were similar in age, gender, race, and education (Table 1). As expected, disease duration was longer in ET-PD than PD. The two groups were similar in terms of CIRS score, CESD-10 score, and total number of prescription medications (Table 1). A higher proportion of PD than ET-PD patients was taking carbidopa–levodopa, although the daily dose (miiligrams) of levodopa was higher in ET-PD (Table 1). A higher proportion of ET-PD than PD patients were taking medications with gamma aminobutyric acid (GABA)-ergic properties (e.g., primidone, benzodiazepines). The Hoehn and Yahr score was I or II in 85% of cases.

**Figure 1:**
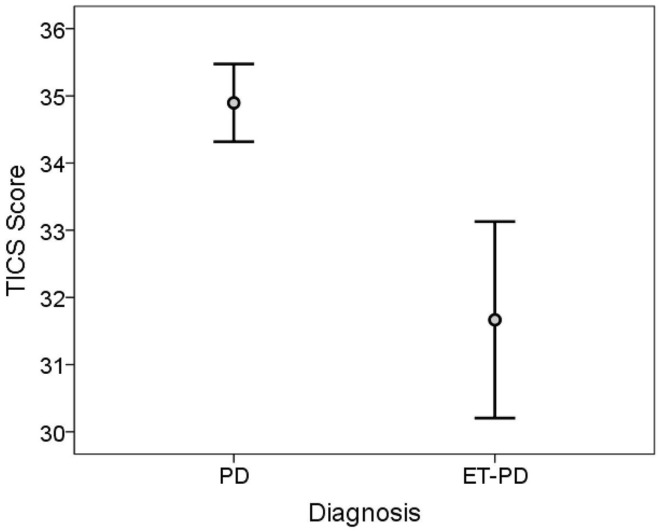
**TICS score in ET-PD vs. PD**. The central circles represent means and the bars represent the 95% confidence intervals.

**Figure 2:**
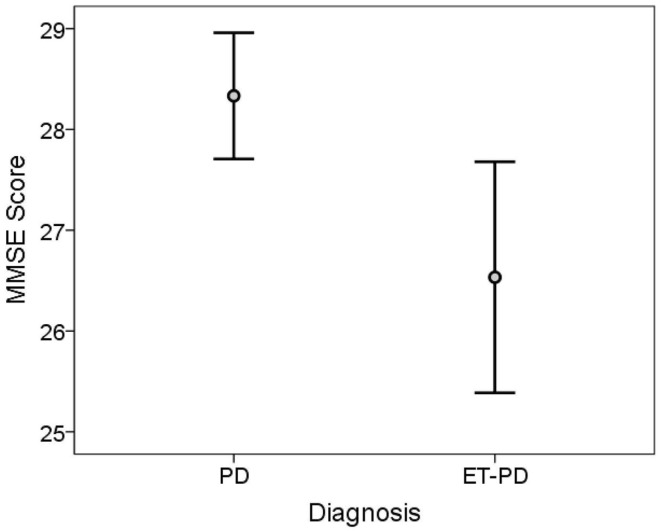
**MMSE score in ET-PD vs. PD**. The central circles represent means and the bars represent the 95% confidence intervals.

The TICS score was lower in ET-PD than PD (*p* < 0.001, Table 2; Figure 1). The MMSE score was lower in ET-PD than PD (*p* = 0.001, Table 2; Figure 2). The orientation subscore was lower in ET-PD than PD (*p* < 0.001, Table 2), as was the language subscore (*p* < 0.001, Table 2) and the working memory subscore (*p* = 0.001, Table 2). The delayed memory subscore was marginally lower in ET-PD than PD (*p* = 0.06, Table 2). The two groups did not differ with respect to the motor/construction subscore (*p* = 0.22, Table 2).

**Table 2:** **Cognitive performance of participants**.

	PD	ET-PD	Significance
*n*	53	30	
TICS score	35.0 ± 2.0 (35.0)	31.7 ± 3.9 (32.0)	*p* < 0.001^a^
MMSE score	28.4 ± 2.2 (29.0)	26.5 ± 3.1 (28.0)	*p* = 0.001^a^
Subscores
Orientation	19.8 ± 0.8 (20.0)	19.1 ± 1.5 (20.0)	*p* < 0.001^a^
Language	9.9 ± 0.3 (10.0)	9.0 ± 1.0 (9.0)	*p* < 0.001^a^
Working memory	14.2 ± 1.5 (15.0)	12.3 ± 3.1 (14.0)	*p* = 0.001^a^
Delayed memory	2.1 ± 1.0 (2.0)	1.8 ± 0.9 (2.0)	*p* = 0.06^a^
Motor/construction	1.8 ± 0.5 (2.0)	1.7 ± 0.6 (2.0)	*p* = 0.22^a^

*Values are mean ± SD (median)*.

*ET, essential tremor; MMSE, Folstein Mini-Mental State Examination; PD, Parkinson’s disease; TICS, telephone interview for cognitive status*.

*^a^Mann–Whitney test*.

**Figure 3:**
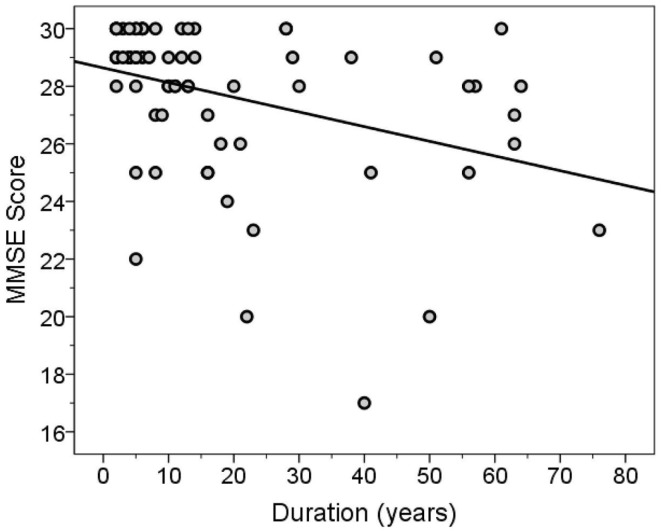
**MMSE score by disease duration (years)**. The regression line is shown.

**Figure 4:**
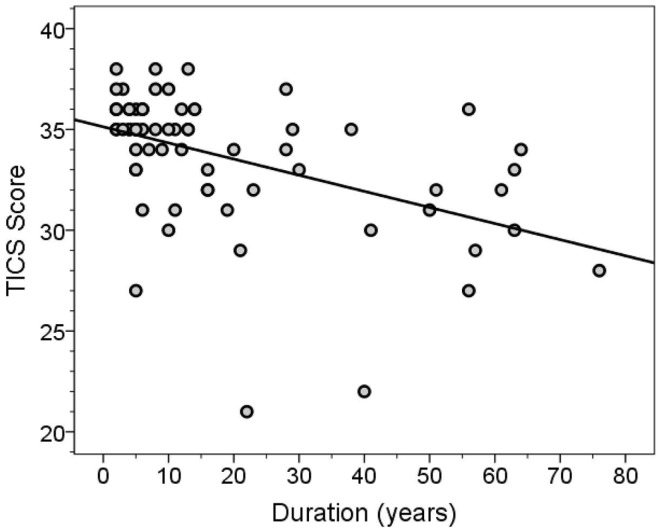
**TICS score by disease duration (years)**. The regression line is shown.

The MMSE and TICS scores were highly correlated (Spearman’s *r* = 0.63, *p* < 0.001). Both scores were inversely correlated with disease duration (for MMSE score, Spearman’s *r* = −0.46, *p* < 0.001, Figure 3; for TICS score, Spearman’s *r* = −0.53, *p* < 0.001, Figure 4) and age (for MMSE score, Spearman’s *r* = −0.36, *p* = 0.001; for TICS score, Spearman’s *r* = −0.40, *p* < 0.001) and were correlated with years of education (for MMSE score, Spearman’s *r* = 0.35, *p* = 0.002; for TICS score, Spearman’s *r* = 0.30, *p* = 0.008). In a linear regression analysis, disease duration (β = 0.07, *p* < 0.001), age (β = 0.13, *p* = 0.047), and education (β = −0.29, *p* = 0.004) were independently associated with transformed TICS score.

Years of education in ET-PD patients was 15.1 ± 4.4 vs. 16.5 ± 2.7 in PD patients (*p* = 0.12, Table 1). To explore the possibility of confounding by education, in a linear regression model, we assessed the association between diagnosis and transformed TICS score (outcome variable), adjusting for years education. In that model, the transformed TICS score remained lower ET-PD than PD patients (β = 0.16, *p* < 0.001), even after adjusting for years of education. A similar linear regression model, examining the association between MMSE and diagnosis, was not possible because the MMSE was not normally distributed.

There was no correlation between Hoehn and Yahr score and cognitive test scores; in ET-PD patients, there was no correlation between total tremor score and cognitive test scores (data not shown).

There was no difference in cognitive scores between ET-PD patients who were taking medication with GABA-ergic properties vs. ET-PD patients who were not taking such medication (for MMSE score, Mann–Whitney = 0.19, *p* = 0.85; for TICS score, Mann–Whitney = 0.85, *p* = 0.40). There was no difference in cognitive scores between ET-PD patients who were taking carbidopa–levodopa vs. ET-PD patients who were not (for MMSE score, Mann–Whitney = 0.56, *p* = 0.58; for TICS score, Mann–Whitney = 0.006, *p* = 0.996). Among patients who were taking carbidopa–levodopa, there was no correlation between daily dose (milligrams) and cognitive test scores (for MMSE score, Spearman’s *r* = −0.18, *p* = 0.18; for TICS score, Spearman’s *r* = −0.18, *p* = 0.19).

To assess whether the group difference in cognitive test scores was an artifact of difference in disease duration, in a sensitivity analysis, we frequency-matched 48 PD and 11 ET-PD patients by both disease duration [9.9 ± 12.5 years (PD) vs. 10.6 ± 5.2 (ET-PD), *t*-test = 0.18, *p* = 0.86]; in these analyses, both cognitive test scores were lower in ET-PD than PD [for MMSE score, 27.3 ± 2.2 (median = 28.0) in ET-PD vs. 28.4 ± 2.2 (median = 29.0) in PD, Mann–Whitney = 2.21, *p* = 0.027; for TICS score, 33.0 ± 2.9 (median = 34.0) in ET-PD vs. 35.0 ± 2.0 (median = 35.0) in PD, Mann–Whitney = 2.17, *p* = 0.03].

## Discussion

As part of a prospective, clinical–epidemiological study, ET-PD and PD patients underwent two tests of global cognition and we tested the *a priori* hypothesis that ET-PD patients would have poorer performance than their counterparts with PD alone. The data, which showed that performance on both cognitive tests was poorer in ET-PD than in PD, supported this hypothesis. That is, the presence of the combined diagnosis seemed to be associated with additive cognitive effects. The difference between the two patient groups was apparent with two distinct cognitive tests, thereby providing construct validity for the results.

These data have a number of clinical implications. The first is that patients with the combined diagnosis may be more likely to experience cognitive difficulty than those with PD alone, and clinicians should be sensitive to this issue. The second implication is that it is possible that this subgroup of patients (i.e., ET-PD) is at greater risk of developing dementia. These data; however, do not directly address this question.

The ET-PD group performed more poorly in several domains (orientation, language, working memory, and marginally in delayed memory). This observed mixture of differences does not simply reflect a greater degree of subcortically driven deficits in the ET-PD group, but also reveals greater impairment in abilities more typically affected by cortical conditions such as Alzheimer’s disease (13). More comprehensive neuropsychological testing is needed to examine the potentially heterogeneous combination of processes that contribute to the cognitive performance issues in patients with the combined diagnosis.

The ET-PD patients had a longer duration of symptoms than the PD patients, as one would have expected – they had had ET for a minimum of 5 years prior to the onset of PD. When we matched the two groups based on duration of symptoms, the difference in cognitive test scores persisted, indicating that symptom duration was not a confounding factor. We did not collect data on duration of PD *per se*; however, the number of years on carbidopa–levodopa did not differ between the ET-PD and PD groups, suggesting that the duration of PD was likely to have been similar in the two groups.

This study should be interpreted within the context of several limitations. First, cognition was evaluated using two brief, global screening instruments rather than a detailed neuropsychological test battery. Despite this limitation, several significant differences were evident; a more detailed cognitive assessment would likely reveal additional differences. Second, the study was designed prior to the widespread use of the Montreal Cognitive Assessment, which is more sensitive to deficits in executive function, and it would be useful in future studies to employ this cognitive screen as well. Finally, we did not collect data on the ON or OFF state of patients and could not assess the potential influence of current motor state on test performance.

This study had several strengths. It is the first to address this particular research question and this specific *a priori* hypothesis, thereby adding another unique data element to our understanding of cognition in ET. Second, a standardized assessment was performed on all study participants by a research worker who was blinded to the *a priori* study hypothesis. Third, participants were enrolled prospectively, thereby avoiding the issue of incomplete data inherent in a retrospective chart review.

In summary, the performance on both cognitive tests and several test subscores was poorer in patients with ET-PD than in PD. That is, the presence of the combined diagnosis, ET-PD, seemed to be associated with additive cognitive effects.

## Author Contributions

EL was involved in the conception and design of this work, the analysis and interpretation of data, the drafting of the manuscript, and he gives final approval of the version to be published and agreement to be accountable for all aspects of the work in question. BR was involved in the conception and design of this work, the acquisition of data, the critical revision of the manuscript, and she gives final approval of the version to be published and agreement to be accountable for all aspects of the work in question. KC was involved in the conception and design of this work, the acquisition of data, the critical revision of the manuscript, and she gives final approval of the version to be published and agreement to be accountable for all aspects of the work in question. SC was involved in the conception and design of this work, the analysis and interpretation of data, the critical revision of the manuscript, and she gives final approval of the version to be published and agreement to be accountable for all aspects of the work in question.

## Conflict of Interest Statement

The authors declare that the research was conducted in the absence of any commercial or financial relationships that could be construed as a potential conflict of interest.
